# Correction: Prevalence and associated factors of playing-related musculoskeletal disorders among music students in Europe. Baseline findings from the Risk of Music Students (RISMUS) longitudinal multicentre study

**DOI:** 10.1371/journal.pone.0258608

**Published:** 2021-10-11

**Authors:** The PLOS ONE Staff

There is an error in affiliation 1 for authors Cinzia Cruder and Marco Barbero. The correct affiliation 1 is: Rehabilitation Research Laboratory 2rLab, Department of Business Economics, Health and Social Care, University of Applied Sciences and Arts of Southern Switzerland, Manno, Switzerland.

There is also an error in affiliation 4 for author Emiliano Soldini. The correct affiliation 4 is: Research Methodology Competence Centre, Department of Business, Health and Social Care, University of Applied Sciences and Arts of Southern Switzerland (SUPSI), Manno, Switzerland.

The publisher apologizes for the error.
